# Insulin/IGF-driven cancer cell-stroma crosstalk as a novel therapeutic target in pancreatic cancer

**DOI:** 10.1186/s12943-018-0806-0

**Published:** 2018-02-23

**Authors:** Ayse Ceren Mutgan, H. Erdinc Besikcioglu, Shenghan Wang, Helmut Friess, Güralp O. Ceyhan, Ihsan Ekin Demir

**Affiliations:** 1 Department of Surgery, Klinikum rechts der Isar, Technical University Munich, München, Germany; 2 Department of Histology and Embryology, Gazi University Institute of Health Sciences, Ankara, Turkey

**Keywords:** IGF-1, Insulin, Pancreatic cancer, Stroma

## Abstract

Pancreatic ductal adenocarcinoma (PDAC) is unrivalled the deadliest gastrointestinal cancer in the western world. There is substantial evidence implying that insulin and insulin-like growth factor (IGF) signaling axis prompt PDAC into an advanced stage by enhancing tumor growth, metastasis and by driving therapy resistance. Numerous efforts have been made to block Insulin/IGF signaling pathway in cancer therapy. However, therapies that target the IGF1 receptor (IGF-1R) and IGF subtypes (IGF-1 and IGF-2) have been repeatedly unsuccessful. This failure may not only be due to the complexity and homology that is shared by Insulin and IGF receptors, but also due to the complex stroma-cancer interactions in the pancreas. Shedding light on the interactions between the endocrine/exocrine pancreas and the stroma in PDAC is likely to steer us toward the development of novel treatments. In this review, we highlight the stroma-derived IGF signaling and IGF-binding proteins as potential novel therapeutic targets in PDAC.

## Background

The latest demographic studies on pancreatic ductal adenocarcinoma (PDAC) indicate that, unless novel diagnostic tools and treatments are developed, PDAC is expected to be the 2nd leading cancer-related cause of death in the United States before 2030 [1].

Smoking, alcohol usage, family history of chronic pancreatitis, male gender, advanced age, high body mass index (BMI) and diabetes mellitus (DM) are risk factors for developing PDAC [2, 3]. Among these, type 2 DM/T2DM has been postulated to be a reason for screening for PDAC, as it frequently precedes the diagnosis of PDAC [4]. Thus, researchers have long wondered whether T2DM is a contributor or a consequence of the PDAC.

Most T2DM patients have hyperinsulinemia, a condition that defines high insulin levels in the blood. Insulin regulates glucose, lipid and amino acid homeostasis, acts on organs such as liver, muscle and adipose tissue lifelong. In a state of hyperinsulinemia, insulin increases bio-availability of another class of factors, i.e. insulin-like growth factors (IGF), which are one of the key regulators of energy metabolism and growth [5].

Due to the plethora of metabolic derangements caused by hyperinsulinemia, researchers have long considered a potentially decisive role for Insulin/IGF signaling in neoplasia, including PDAC [6]. Although the first results of the clinical trials with compounds that target insulin/IGF signaling in PDAC have been disappointing, researchers have recently directed their research towards understanding the role of Insulin/IGF-1R signaling in the *cross-talk between cancer cells and stroma*. Indeed, there is still a major knowledge gap in how exactly the dynamic stroma of PDAC can affect the complex endocrine and exocrine compartments of the pancreas. Thus, enlightening the insulin/IGF-driven interaction between cancer cells, endocrine pancreas, and the stroma may be key to understanding the progression of PDAC and of PDAC-associated diabetes, and thereby open the door to the development of efficent therapies that target cancer cells and tumor stroma at the same time. In this review, we summarize the role of Insulin/IGF signaling pathway in the reciprocal interactions of stromal cells with cancer cells in the PDAC microeinvironment and suggest a research line to that may create opportunities to develop novel treatments for PDAC.

### Tumor cell-intrinsic effects of IGF signaling in PDAC

**Fig. 1:**
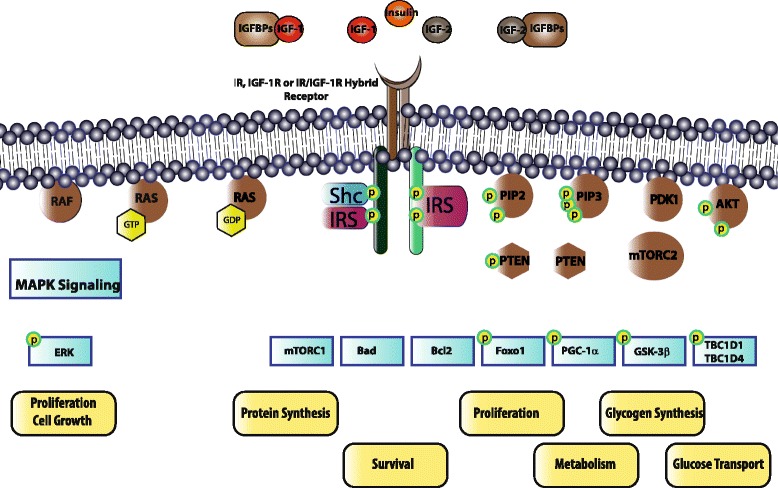
Overview of the intracellular signaling pathways that are linked to Insulin/IGF signaling in PDAC. Insulin, IGF-1 and IGF-2 can bind to IRs (IR-A and IR-B), IGF-1R and IR/IGF-1R hybrid receptors. Homodimer IR-A receptor has higher binding affinity to IGFs compared to the homodimer IR-B receptor. IGF-1R has binding affinity to IGF-1, IGF-2 and insulin. IGF-1 is the dominant ligand of IGF-1R. Insulin has a stronger binding affinity to IGF-1R compared to IGF-2. IGF-2R has binding affinity only for IGF-2. IGF-1R can form hybrid receptor with either IR-A or IR-B. Hybrid receptors have higher affinity to IGF-1 and IGF-2 ligands compared to the insulin. Thus, IGF-2R can decrease the bioavailability of IGF-2 in the circulation and attenuates insulin/IGF signaling axis by clearance of the circulating IGF-2. When insulin and IGFs that are free from IGFBPs bind to IR, IGF-1R or IR/IGF-1R hybrid receptors, these receptors are autophoshorylated. Depending on the expression level of the receptors in different tissues, autophosphorylation of these receptors activates different signaling pathways. Autophosphorylation of these tyrosine kinase receptors phosphorylates adaptor proteins such as insulin receptor substrate (IRS) and Shc. IRS phosphorylation initiates phosphatidylinositol-3-kinase (PI3K)-Protein Kinase B (PKB)/AKT pathway. On the other hand, binding of adaptor proteins such as Shc to the phosphorylated receptors or to the IRS initiates mitogen activated protein kinase (Ras-MAPK) signaling pathway. To activate the PI3K-PKB/AKT pathway, the second messenger PIP3 should be generated by adding a phosphate group to PIP2. Phosphorylation of PIP2 to PIP3 is reversible, and dephosphorylation of PIP3 is regulated by the tumor suppressor PTEN. Membrane bound PIP3 triggers activation of the PDK1 protein, which phosphorylates AKT. To be fully activated, AKT should be phosphorylated not only by PDK1 but also by mTORC2. Activation of AKT induces many different effects such as recruitment of glucose transporters to the membrane, glycogen synthesis, lipid synthesis, protein synthesis, metabolism, cell survival and apoptosis. Activation of the Ras-MAPK signaling pathway is the second pivotal role of insulin/IGF signaling axis. To activate Ras-MAPK signaling pathway, first the adaptor proteins should bind to other adaptor proteins to activate guanine nucleotide exchange factor (GEF) of Ras. Conversion of Ras-GDP to Ras-GTP by GEF activates Ras protein. Activation of Ras leads to the induction of MAPK signaling cascade that regulates cell proliferation

Insulin and IGF are closely related and conserved systemic growth factors that are produced by different organs. Insulin is produced by β-cells of the pancreas, and IGF ligands IGF-1 and IGF-2 are produced by the liver in response to growth hormone (GH) stimulation that is secreted from the anterior pituitary gland [6]. Insulin (IR) and IGF (IGFR) receptors belong to the receptor tyrosine kinase (RTK) family. There are two different insulin receptors and two different IGF receptors, IR-A/IR-B and IGF-1R/IGF-2R, respectively. IGF-1R is expressed nearly in all tissues [7, 8]. Moreover, 40–90% of the IGF-1R on tissues are found to be IGF-1R/IR hybrid receptors [7, 8]. Such hybrid receptors display higher binding affinity to IGF ligands compared to insulin [9]. IGF-2R is ubiquitously expressed, and yet IGF-2R receptor activation does not induce activation of insulin/IGF signaling axis [6]. IGF-2R can only bind to IGF-2, but insulin, IGF-1 and IGF-2 can bind to IR, IGF-1R and IR/IGF-1R hybrid receptors with varying binding affinity (Fig. 1) [10–12]. Thus, the crosstalk between insulin and IGF signaling axis designates the complexity of this signaling pathway and its numerous modes of activation.

IGF-1 and IGF-1R are known to be abundantly expressed in the PDAC tissue, and activated Insulin/IGF signaling in PDAC cells was found to regulate the basal growth rate of the cancer cells [13, 14]. In fact, IGF-1R expression is correlated with higher tumor grade, and its co-expression with epidermal growth factor receptor (EGFR) was shown to be be significantly associated with poor survival in PDAC [15]. IGF-2R mRNA and protein levels were previously shown to be upregulated in human pancreatic cancer tissues, particuarly in the nucleus of ductal-like pancreatic cancer cells, when compared to the normal pancreatic tissue [16]. When the inability of IGF-2R to induce Insulin/IGF signaling is considered, IGF-2R seems to decrease the bioavailability of IGF-2 in the circulation and attenuates insulin/IGF signaling axis by clearance of the circulating IGF-2.

IGF signaling pathway further consists of six IGF binding proteins (IGFBPs) and 10 IGFBP-related proteins (IGFBP-rPs) [17]. In circulation, IGFs are found in a protein-bound form with IGFBPs [18], protecting the ligands from degradation, and extending the half-life and stability of the circulating IGFs [17, 19]. Free IGFs have a higher binding affinity to IGFBPs than to IGF-1R, IR and IR/IGF-1R hybrid receptors. Therefore, distribution of IGFs in tissues and attenuation of the Insulin/IGF signaling is regulated by IGFBPs, which regulate the bio-availability of IGFs and contribute to attenuation of the Insulin/IGF signaling axis [17, 19, 20].In the serum and in the pancreas of PDAC patients, IGFBP-1, IGFBP-3, IGF-1 and IGF1R are over-expressed [21]. In addition, high IGF-1 levels in the serum of PDAC patients are associated with high levels of IGFBP-3 again in the serum when compared to healthy individuals [22]. Besides, increase in the serum levels of IGFBP-3 seems to be associated with the risk of death from PDAC [22]. Recent studies highlighted that high IGF-1/low IGFBP-3 concentrations might be associated with increased PDAC risk [23, 24]. Accordingly, patients with high IGF-1R/low IGFBP-3 expression in the pancreas are diagnosed with advanced PDAC and exhibit overall poor survival [25]. This observation suggests that IGF-1/IGFBP-3 expression levels might be altered during the progression of PDAC, but, most importantly, elevated levels of free IGF-1 together with IGF-1R expression is correlated with poor prognosis and survival. In addition, IGFBP-3 is one of the p53 response genes and is involved in p53-induced apoptosis independent of IGF-1 signaling [26]. Interestingly, p53 can also directly modulate IGF-1R expression by regulating the IGF-1R gene promoter [27].Therefore, inactivation or altered expression of tumor suppressors such as p53 might be another reason for the overexpression of IGF-1R receptors in PDAC.

### IGF signaling in the PDAC stroma

**Fig. 2:**
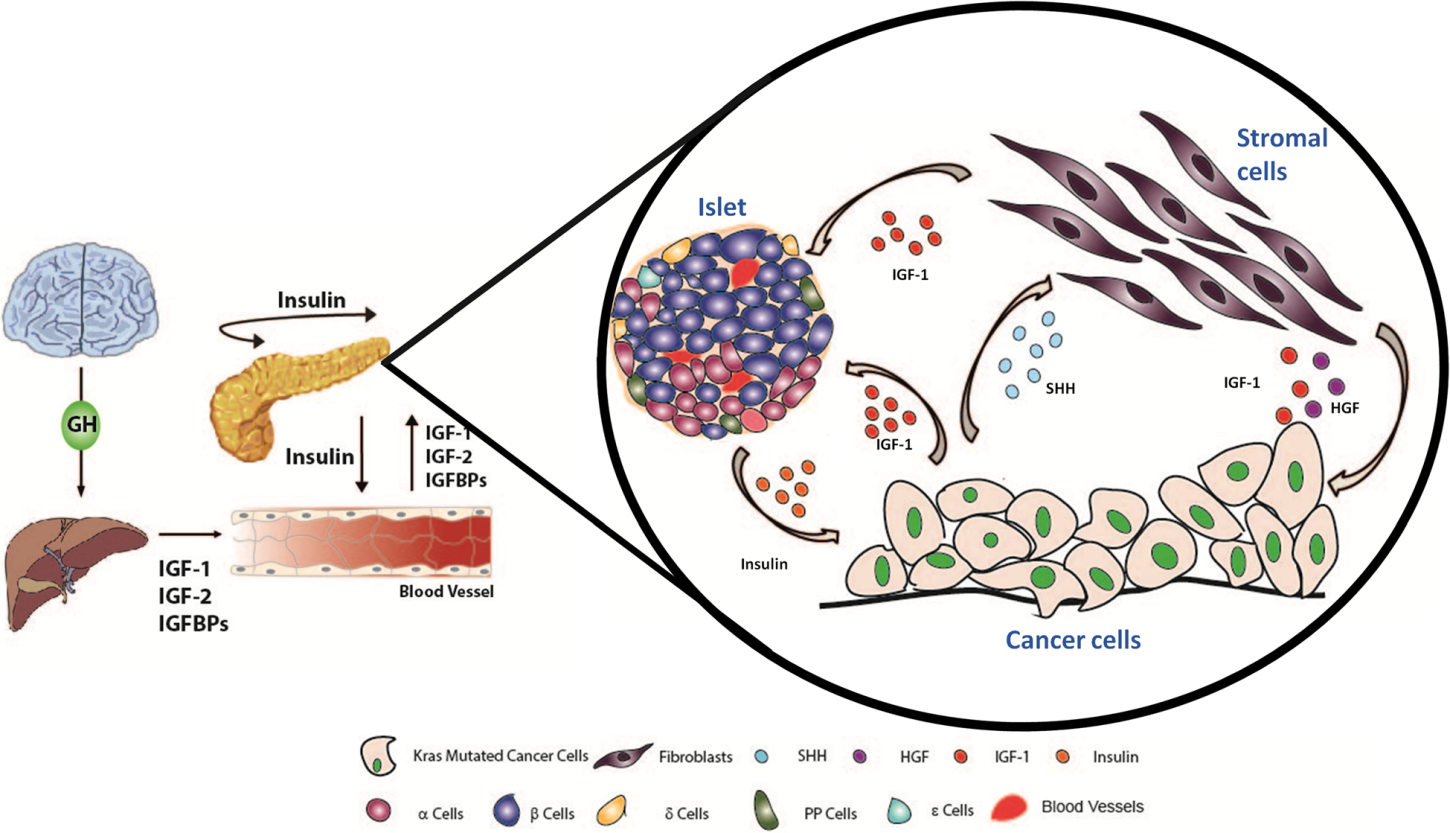
Under-recognized molecular interactions of IR-, IGF-1R- and IR/IGF-1R- expressing pancreatic cancer cells and stromal cells. IGF ligands and IGFBPs are produced by the liver in response to growth hormone (GH) secretion from the anterior pituitary gland. IGF ligands and IGFBPs circulate in the body, and when free IGFs reach the tissues such as the pancreas that express IR, IGF-1R or IR/IGF-1R receptors, they induce activation of the target signaling pathways. Endocrine part of the pancreas secretes insulin, which is also involved in the regulation of IGFs that are secreted by the liver. Insulin does not only affect the pancreas in an autocrine and paracrine manner, but also regulates energy metabolism of the body. In PDAC, IR, IGF-1R and IR/IGF-1R receptors and IGF-1 are overexpressed in cancer cells. Moreover, cancer cells that harbor the mutated oncogenic Kras pre-dominantly secrete sonic hedgehog (Shh) that activates (myo-) fibroblasts that reside in the tumor stroma. Activated (myo-) fibroblasts that secrete IGF-1 in response to Shh activation induce IGF-1R signaling on cancer cells. Moreover, secreted proteins of activated fibroblasts, e.g. hepatocyte growth factor/HGF and CCL5, augment the migration of cancer cells and anti-tumor immunity via IGF-1R on cancer cells. On the other hand, the actual impact of altered IGF-1 levels and enhanced Insulin/IGF-1R signaling in PDAC tumor stroma, and on the endocrine β-cell function is yet to be discovered. Targeting stroma-derived IGF signalling, but also the levels of IGFBPs, can be novel tailored therapy options in PDAC

**Fig. 3:**
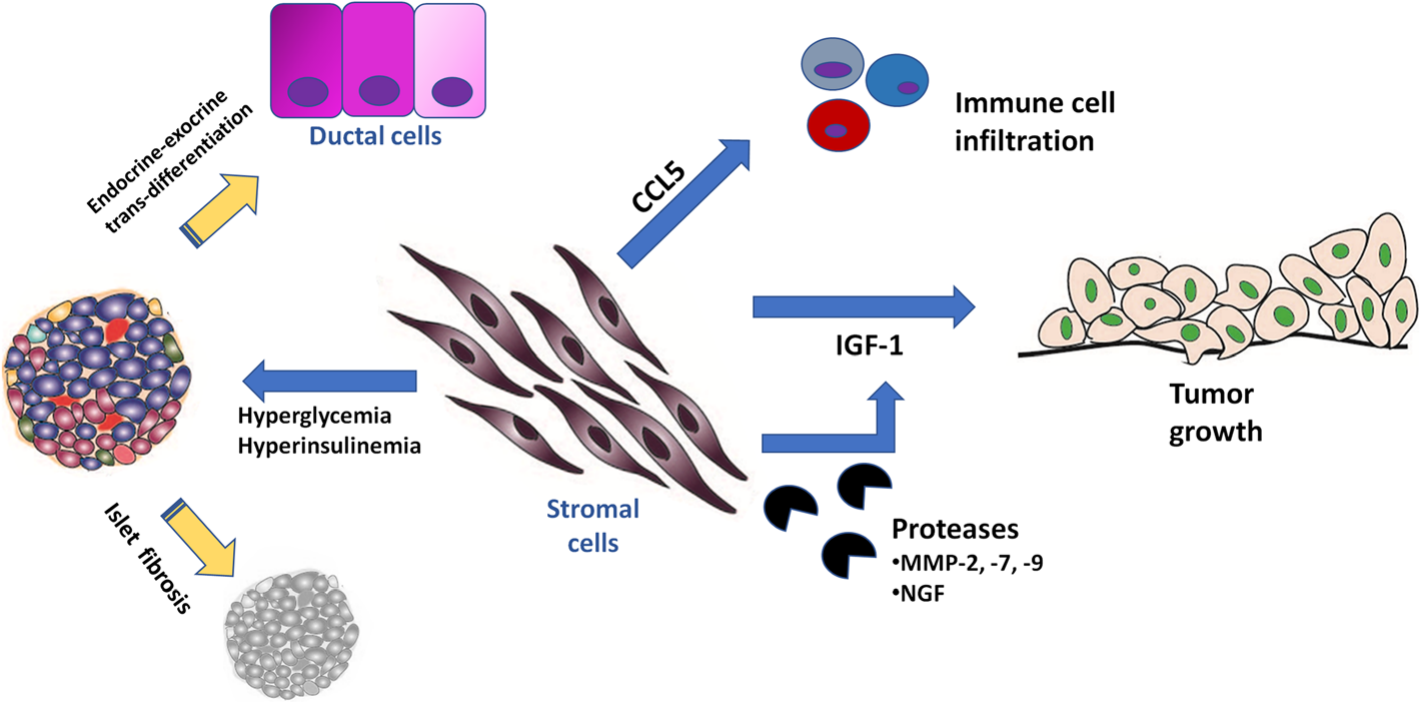
Five lines of evidence supporting a key role for stroma-derived IGF signaling in PDAC. 1) Stromal cells such as fibroblasts or pancreatic stellate cells (PaSCs) can secrete IGF-1 and enhance the migration capacity of PDAC cells. 2) Proteases such as MMP-3, MMP-7, or MMP-9 that are secreted by stromal cells can cleave IGFBPs and thereby fine-tune IGF activity. 3) Via chemokine secretion, stromal cells can chemo-attract immune cells such as macrophages to the tumor microenvironment and can thereby influence the local tumor control. 4) In a hyperglycaemic state that frequently accompanies PDAC, stromal cells can become activated and cause islet fibrosis and thereby aggravate the hyperglycaemic state. 5) Increasing tissue damage and fibrosis can lead to recruitment of endocrine or exocrine progenitors that can result in transdifferentiation between these cell types

Despite the presence of several studies that demonstrated the effects of activated IGF signaling on cancer cells in PDAC, the impact of stromal IGF signaling or stroma-derived IGFs in PDAC has been recognized only very recently. PDAC cells are typically surrounded by a dense stroma, which is classically assumed to serve as a protective barrier against tumor spread [28, 29]. The majority of tumor stroma in PDAC is composed of “acellular” components. i.e. extracelluar matrix proteins such as collagen, fibronectin or laminin, The “cellular” stroma contains the key actors of the tumor microenvironment such as immune cells, endothelial cells, pancreatic stellate cells, fibroblasts, or neural cells. There are five lines of evidence that suggests a key role for stromal IGF signaling in the progression of PDAC (Figs. 2, 3).

#### Activated stromal (myo-) fibroblasts as a leading source of IGF-1 in PDAC

In more than 90% of PDAC cases, KRAS is over-activated by mutations [30]. KRAS^(G12D)^-mutated cancer cells activate stromal fibroblasts via Sonic Hedgehog (Shh) pathway [31]. This activation does not only provide survival signals for the fibroblasts, but also activates IGF-1R on cancer cells via IGF-1 that is secreted by fibroblasts and by pancreatic stellate cells (PaSCs) in response to Shh (Fig. 2) [31–33]. Indeed, current evidence suggests that stromal cells may be the foremost source of IGF-1 in the PDAC microenvironment. IGFs that are secreted from stromal cells can act on cancer cells via direct IGF-1R signaling, and together with hepatocyte growth factor/HGF, can phosphorylate Annexin A2/AnxA2, a protein that has a well-established role in invasion/metastasis [33].

In addition to Shh, stromal (myo-) fibroblasts can also become activated under tumor hypoxia. Indeed, PDACs are hypovascularized and thus hypoxic tumors [34]. In PDAC, cancer-associated fibroblasts produce IGF-1 under hypoxic conditions and promote tumor cell migration via IGF-1R signaling under hypoxia in vitro [34]. Remarkably, the migration capacity of tumor-derived PaSCs is also prominently greater both at basal conditions and after IGF-1 stimulation [35]. One molecular reason for this may be the expression levels of IGFBP in PaSCs: Tumor PaSCs have lower expression levels of IGFBP-3 and higher expression levels of IGFBP-2 compared to the normal PaSCs. Considering the greater migratory capacity of tumor PaSCs when compared to normal PaSCs, the reduction in IGFBP-3 levels seems to overweigh the elevation of IGFBP-2, resulting in a net increase in the IGF-1 availability [35].

#### Control of IGFBP levels by stromal proteases

Expression levels of the regulatory IGFBPs in the liver and concentration of these proteins in circulation or in the tumor tissue are subject to modulation via proteases [36]. Importantly, the desmoplastic pancreatic cancer stroma contains many different proteases. Such proteases may cause degradation of IGFBPs and lead to increased amounts of free IGFs within the tissue. For instance, nerve growth factor (NGF), a family member of kallikrein proteases, can degrade IGFBP-3, IGFBP-4 and IGFBP-6 and is strongly upregulated in PDAC [37, 38]. Similarly, cathepsin D, which also displays high expression levels in PDAC patients compared to healthy individuals, can mediate proteolysis of IGFBPs [39, 40]. Likewise, IGFBP-1, − 2, − 3, − 4 and - 6, are substrates of MMP-2, MMP-7 and MMP-9 proteases, which are expressed in the peritumoral stroma and cancer cells in PDAC [41–44]. Direct modulation of IGFBPs at protein level via stromal proteases can be considered as one of the sources of activated IGF signaling in PDAC.

#### Regulation of anti-tumor immunity in PDAC stroma by IGF signaling

The dynamic stroma can also regulate anti-tumor immunity [45]. Wide range of studies have implied the involvement of CCL5/CCR5 signaling axis in anti-tumor immunity, invasion and metastasis [46]. Interestingly, IGF-1 maintains secretion of CCL5 from stromal cells, in particular mesenchymal stem cells that are in physical contact with PDAC cancer cells in vitro, resulting in the recruitment of tumor-targeting immune cells [47]. Importantly, active signalling through IGF-1R is needed for this cross-talk [46, 47]. Like pancreatic myofibroblasts, tumor associated macrophages (TAMs) are the other stromal source of IGF ligands [48]. Recently, it has been found that TAM infiltration in PDAC patients is correlated with increased IR/IGF-1R expression, and inhibition of IR/IGF-1R axis in preclinical diseases models improves chemotherapy responses [48].

#### Hyperinsulinemia promotes stromal activation and fibrosis in PDAC

Western-type high fat diet can result in, hyperinsulinemia, and over time lead to hyperglycemia due to insulin resistance, and particularly to elevated IGF-1 levels in the circulation. This metabolic derangement was shown to activate PaSCs that express IR-A and IGF-1 receptors [49], and to boost stromal fibrosis and also specifically fibrosis within islets, which is typically encountered in T2DM [50, 51].

#### Impact on the exocrine-endocrine crosstalk in PDAC

Previous studies suggested that insulin/IGF signaling can affect both the exocrine and endocrine compartment of the pancreas. In the *exocrine* compartment, IGF-1 is mainly responsible for acinar cell regeneration and regulation of amylase synthesis [52]. Indeed, IGF-1 that is secreted from fibroblasts was shown to promote acinar cell recovery during acute pancreatitis [53]. Besides, IGF-1 can reduce the tissue damage due to caerulein-induced pancreatitis [54]. Moreover, after partial pancreatectomy in mice, acinar cell proliferation was linked to IGF-1 presence in the microenvironment and was hampered in aging mice due to the loss of responsiveness to IGF-1 [55, 56].

The impact of Insulin/IGF signalling on development and function of the endocrine pancreas has been extensively studied and summarized before [57–59]. In the *endocrine* compartment, IGFs, IGF-1R and IGFBPs were shown to control the function of β-cells. IGF-1 can stimulate β-cell proliferation and increase β-cell mass, increase basal insulin production regardless of mass proliferation [60–63]. Furthermore, low levels of circulating IGF-1 reduces β-cells function [64]. On the other hand, IGF-2 ligand overexpression has been found to damage the function of β-cells in vivo [65]. Interestingly, IGFBP-3 also affects β-cells. In vitro studies suggested that IGFBP-3 can trigger apoptosis in insulin-secreting cells [66]. Moreover, IGFBP-3 is able to regulate insulin secretion from β-cells in response to glucose, in vivo [67].

Substantial evidence suggests that both endocrine islets and exocrine pancreas tissue can modulate each other’s function. Earlier studies that had been conducted with mouse models of type I diabetes mellitus, disclosed that hormone secretion from islets modulate the structure and the functionallity of exocrine cells in the pancreas [68–71]. Interestingly, in mouse models of type II diabetes mellitus and in diabetic patients, extracellular matrix in-between islets and acinar cells is frequently lost [72]. Tissue fibrosis and pericapillary fibrosis in the islets lead to loss of cell to cell communication between islets and acinar cells [72]. Thus, this phenomenon may not only alter the trophic effects of the endocrine cells on the exocrine cells, but also diminish the efficent use of digestive enzymes by gut and thereby cause maldigestion [72]. Besides, it is also imaginable that alterations in IGFBP levels in the PDAC stroma can be indirectly responsible for loss of islets and emergence of diabetes and maldigestion in PDAC. Of note, one should consider that endocrine β-cells that express oncogenic K-ras can also be one potential progenitor for PDAC under chronic tissue inflammation [73]. Overexpression of transcription factors that normally control endocrine differentiation during embryonic development (i.e., Neruogenin 3, Pax6, MafA, Pdx1) in ductal cells can lead to exocrine-endocrine differentiation [74–76]. Moreover, ductal cells are able to undergo ductal-endocrine differentiation in the presence of proinflammotary cytokines such as TNF-α, IL-1β and IFN-γ via STAT3 activation [77]. Interestingly, endocrine progenitors like Sox9 (+) /Pdx1 (+) /Ngn3(+) cells are found in the intercalated ducts of adult pancreata [78]. Moreover, patients with chronic pancreatitis were reported to have insulin-expressing ‘*islet progenitors’* on their ducts [79]. Whether such cells are present in the PDAC stroma has yet to be invesigated. The function of these insulin-expressing cells on pancreatic ducts in the normal and diseased pancreas is also currently unknown. Moreover, the potential role of insulin-secreting endocrine cells in the progression of PDAC and the impact of PDAC tumor microenvironment on insulin-secreting endocrine cells is yet to be discovered. Such a “three-way”, insulin/IGF-driven interaction between exocrine/cancer cells, endocrine pancreas, and the stroma may be key to understanding the progression of PDAC and of PDAC-associated diabetes (Fig. 3).

### The impact of insulin/IGF-1R signaling on chemotherapy and targeted therapies in PDAC: current and novel directions

PDAC is frequently resistant to the current chemotherapy regimens. Recently, FOLFIRINOX, a novel chemotherapy regimen containing four different chemotherapy drugs (follinic acid, fluorouracil, oxaliplatin and irinotecan) was reported to increase the overall survival of patients with unresectable metastatic PDAC to 11.1 months from 6.8 months, a success rate that is far from being satisfying [80]. This fact points out the urgent need of developing novel treatment strategies or novel targeted therapeutics.

There is accumulating evidence suggesting that IGF-1R pathway inhibitors may enable conceivable benefits in PDAC treatment. Aiming to overcome chemotherapy resistance and to develop better adjuvant therapies within the last decade, many different small molecule inhibitors/monoclonal antibodies against IGF-1R and neutralizing antibodies against IGF ligands have been developed and tested in pre-clinical studies. Even though promising results were obtained with the developed compounds during in vitro, during pre-clinical studies in vivo, and at Phase I/Phase II clinical trials, the overall outcome of advanced clinical trials is yet disappointing. Table 1 provides a comprehensive overview of clinical trials that target insulin/IGF signalling in pancreatic cancer (Table 1).

**Table 1:** Summary of clinical trials that target insulin/IGF signalling in PDAC

Author/Principal investigators	Malignancy	Treatment	NCT accession number	Enrollment number	Phase	Status	Study type	Primary outcome/objectives	Summary of results
Kindler, H.L. et al. [86]	Metastatic pancreatic cancer	Drug 1: Placebo+Gemcitabine Drug 2: Ganitumab (a mAb antagonist of IGF-1R) + Gemcitabine	NCT00630552	42	II	Completed	Randomized Open label Placebo controlled	To evaluate the efficacy and safety of Ganitumab/ Gemcitabine treatment in patients with metastatic pancreatic cancer	A slight improvement in 6-month survival rate in patients who are treated with Ganitumab/Gemcitabine compared to the patients who have received Gemcitabine monotherapy has been observed
Fuchs, C.S. et al. [82]	Metastatic pancreatic adenocarcinoma	Drug 1: Placebo+Gemcitabine Drug 2: Ganitumab+Gemcitabine	NCT01231347	825	III	Terminated	Randomized Double blind Placebo controlled Multicenter	To evaluate the efficacy and safety of Ganitumab/Gemcitabine in first-line treatment of metastatic pancreatic adenocarcinoma	No improvement in the survival rate of patients that are treated with Ganitumab/Gemcitabine compared to the patients that received Gemcitabine monotherapy
Tabernero, J. et al. [87]	Advanced, refractory solid tumours including pancreatic cancer	Drug 1: Ganitumab Drug 2: Conatumumab. (mAb that binds to DR5)	NCT00819169	89	Ib-II	Terminated	Non-randomized Open label Parallel assignment	Phase Ib: To determine the dose of Ganitumab/Conatumumab treatment. Phase II: To evaluate the efficacy of the combined Ganitumab/ Conatumumab treatment in the patients with pancreatic, lung, colorectal, ovarian cancers and sarcoma	Ganitumab/Conatumumab treatment is safe to apply but has no effects on survival rate of patients in the tested population
Philip, P.A et al. [88]	Stage IV pancreatic cancer	Drug 1: Cixutumumab (mAb antagonist of IGF-1R) Drug 2: Erlotinib (EGFR Inhibitor) Drug 3: Gemcitabine	NCT00617708	134	Ib-II	Completed	Randomized Open label Parallel assignment	Phase Ib: To determine the dose of Cixutumumab to be used in combination with Erlotinib/Gemcitabine Phase II: To evaluate the efficacy of Cixutumumab/ Erlotinib/ Gemcitabine in patients with pancreatic cancer	No difference in progression free survival of patients who received Cixutumumab/Erlotinib/Gemcitabine treatment compared to the patients that are treated with Erlotinib/Gemcitabine
Javle, M. et al.	Pancreatic adenocarcinoma	Drug 1: MK-0646 (Dalotuzumab- mAb, IGF-1R antagonist) Drug 2: Gemcitabine Drug 3: Erlotinib	NCT00769483	100	I-II	On-going, not recruiting participants	Randomized Open label Parallel assignment	Phase I: To determine the ‘maximum tolerated dose (MTD)’ of MK-0646/ Gemcitabine, MK-0646/ Gemcitabine/Erlotinib and Gemcitabine/Erlotinib combined therapy Phase II: To evaluate the ‘progression free survival’ under the three-different therapies with the MTD determined in Phase I	Results are expected by November 2018
Braghiroli, M.I. et al. [89]	Advanced metastatic pancreatic cancer	Drug 1: Paclitaxel Drug 2: Metformin	NCT01971034	41	II	Completed	Open label Single group assignment	To evaluate efficacy of Metformin/Paclitaxel treatment compared to the standard Paclitaxel monotherapy	Combined therapy was poorly tolerated by patients and did not improve state of the disease in patients
Renouf, D.J. (British Columbia Cancer Agency)	Respectable PDAC	Drug 1: Metformin	NCT02978547	20	II	On-going, not open yet to recruit participants	Open label Single group assignment	To evaluate the effect of neoadjuvant metformin treatment on tumor cell growth	Results are expected by January 2019
Merrimack Pharmaceuticals	Metastatic pancreatic adenocarcinoma	Drug 1: MM-141 Drug 2: Placebo Drug 3: Gemcitabine Drug 4: Nab-Paclitaxel	NCT02399137	260	II	On-going, recruiting participants	Randomized Double blind Placebo control Parallel assignment	To evaluate the efficiency of MM-141/Nab-Paclitaxel/Gemcitabine combined therapy compared to the Nab-Paclitaxel/Gemcitabine therapy	Results are expected by November 2018
Yeh, J. (John Hopkins University)	Solid tumors including pancreatic cancer	Drug 1: Metformin Behavioral 1: Coach-directed behavioral weight loss Behavioral 2: Self-control weight loss	NCT02431676	120	II	On-going, recruiting participants	Randomized Single blind Parallel assignment	To evaluate the IGF-1 levels and IGF-1/IGFBP-3 ratio in the serum of participants within the next 6 and 12 months survival after surgery.	Results are expected by June 2018
Suleiman, Y. et al. [90]	Advanced or metastatic pancreatic cancer	Drug 1: SOM 230 LAR (somatostatin agonist and potent IGF-1R inhibitor) Drug 2: Gemcitabine	NCT01385956	20	I	Completed	Open label Single group assignment	To evaluate the safety and tolerability of SOM 230 LAR/Gemcitabine treatment	Treatment is well tolerated

Insulin/IGF-1R signaling is one of the signaling pathways that govern the sensitization of cancer cells to gemcitabine [48, 81]. Secreted IGFs from activated myofibroblasts directly act on IGF-1R, which promotes resistance to gemcitabine in pre-clinical studies [48, 81]. However, combined administration of gemcitabine with ganitumab, a monoclonal antibody that inhibits IGF-1R activity, did not show any significant improvement in the survival of PDAC patients (Table 1) [82]. In another strategy, gemcitabine in combination with cetuximab, an epidermal growth factor receptor (EGFR) inhibitor, also failed to show any improvement in survival during Phase II/Phase III trials [83]. Recently, MM-141, a tetravalent bispecific antibody that recognizes IGF-1R and EGFR family member ErbB3, provided promising results in pre-clinical studies [84]. Currently, MM-141 is being tested in Phase II clinical trials in combination with Nab-paclitaxel plus gemcitabine (Table 1, NCT02399137). As another approach, researchers have been testing the effect of another IGF-1R monoclonal antibody, MK-0646, in combination with gemcitabine and erlotinib, an RTK inhibitor that targets EGFR (Table 1, NCT00769483). Even though dual inhibition of IGF-1R/EGFR sounds promising, one should remember that IGF-1R and IR show high homology. Hence, even if IGF-1R receptors are inhibited together with other receptors that promote PDAC progression, IR receptors and hybrid IR/IGF-1R receptors that show strong resemblance to IGF-1R, can be still active and take over the function of the blocked IGF-1R receptor in the presence of insulin or IGFs. Blockade of insulin receptors may have wide-ranging systemic side effects, such as hyperglycemia. One way to overcome this problem is to use specific neutralizing antibodies against IGF-1/IGF-2. Indeed, in pre-clinical studies, cancer proliferation and tumor-promoting effects of mTOR signaling are reduced by BI836845, a neutralizing antibody against IGFs [85]. Phase I clinical trials of this agent in combination with other drugs in non-small-cell lung cancer and prostate cancer are ongoing (NCT02191891 and NCT02204072, respectively). However, studies that investigate the efficacy of this agent in PDAC currently do not exist.

Multiple trials testing therapy regimens that combine conventional cytotoxic drugs with molecules that target IGF-1R signaling have failed to show a major impact on the natural progression of PDAC. Although dual inhibition of IGF-1R signaling together with another RTK that is involved in PDAC progression is the current focus, it is crucial to choose the best partner to inhibit. Moreover, most of the compounds that target IGF-1R signaling either inhibit specifically IGF-1R activation or neutralize IGF-1 and IGF-2. Researchers have not known whether neutralizing antibodies are efficient and completely block the side effects of elevated levels of IGFs in the tissue. Besides, the source and the function of the increased amounts of IGFs in cancer stroma has only been recently identified. Pancreas is a complex tissue, and we should build on our knowledge on the impact of enhanced IGF-1R signaling on the “three-way”, insulin/IGF-driven interaction between cancer cells, endocrine pancreas, and the stroma.

Based on the knowledge depicted above, we propose that 1) specific targeting of stroma-cell-derived IGFs, and 2) targeting the levels of stroma-derived IGFBPs can have more promising traits in the therapy of PDAC. Focusing more on this phenomenon can help researchers to identifty novel therapy targets for dual inhibition IGF-1R signaling, and other signaling pathways, and thereby can create an oppurtunity for dual targeting of stroma and cancer cells.

## Conclusion

Insulin/IGF-1R signaling axis is one of the multiple dysregulated pathways in PDAC, and its roles in the progression of PDAC seem to be multifold. Although there are enormous efforts to develop novel targeted therapies against this signaling axis, recent clinical trials have not been successful. Evidence from cancers other than PDAC suggest that usage of neutralizing agents against IGF-1/IGF-2 can be a promising approach. However, an under-investigated niche is the role of stromal insulin/IGF-1 signaling and the contribution of IGFBPs. Tailored, targeted therapies against *stromal* insulin/IGF-1 signaling can have beneficial effects both on cancer progression and the deregulated endocrine function. Thus, targeting the stromal activity of this pathway may be a novel, viable option in the future treatment of PDAC.
